# Brain Damage in Preterm and Full-Term Neonates: Serum Biomarkers for the Early Diagnosis and Intervention

**DOI:** 10.3390/antiox12020309

**Published:** 2023-01-29

**Authors:** Serafina Perrone, Federica Grassi, Chiara Caporilli, Giovanni Boscarino, Giulia Carbone, Chiara Petrolini, Lucia Maria Gambini, Antonio Di Peri, Sabrina Moretti, Giuseppe Buonocore, Susanna Maria Roberta Esposito

**Affiliations:** 1 Neonatology Unit, Pietro Barilla Children’s Hospital, Department of Medicine and Surgery, University of Parma, 43126 Parma, Italy; 2 Pediatric Clinic, Pietro Barilla Children’s Hospital, University of Parma, Via Gramsci 14, 43126 Parma, Italy; 3 Department of Molecular and Developmental Medicine, University of Siena, 53100 Siena, Italy

**Keywords:** intraventricular hemorrhage, periventricular leukomalacia, cerebral palsy, hypoxic ischemic encephalopathy, neonatal stroke, oxidative stress, neurodevelopment, cerebral damage, S-100B

## Abstract

The Brain is vulnerable to numerous insults that can act in the pre-, peri-, and post-natal period. There is growing evidence that demonstrate how oxidative stress (OS) could represent the final common pathway of all these insults. Fetuses and newborns are particularly vulnerable to OS due to their inability to active the antioxidant defenses. Specific molecules involved in OS could be measured in biologic fluids as early biomarkers of neonatal brain injury with an essential role in neuroprotection. Although S-100B seems to be the most studied biomarker, its use in clinical practice is limited by the complexity of brain damage etiopathogenesis and the time of blood sampling in relation to the brain injury. Reliable early specific serum markers are currently lacking in clinical practice. It is essential to determine if there are specific biomarkers that can help caregivers to monitor the progression of the disease in order to active an early neuroprotective strategy. We aimed to describe, in an educational review, the actual evidence on serum biomarkers for the early identification of newborns at a high risk of neurological diseases. To move the biomarkers from the bench to the bedside, the assays must be not only be of a high sensitivity but suitable for the very rapid processing and return of the results for the clinical practice to act on. For the best prognosis, more studies should focus on the association of these biomarkers to the type and severity of perinatal brain damage.

## 1. Introduction

Multiple factors acting in the pre- or post-natal period (i.e., metabolic complications, nutritional intake, toxic, and infectious disease) could influence the pathophysiology of cerebral damage [1]. Many studies have been conducted to explain the fetal-neonatal perturbations that predispose to the genesis of a neurological delay or other diseases in adult life [2]. Maternal diabetes, prenatal hypoxic/ischemic events, inadequate nutritional intake in early life, early metabolic complications, inflammatory/infective insults, and transfusions are all specific triggers for the activation of oxidative stress (OS), with a rapid increase in the generation of free radicals (FRs). Therefore, these factors could be triggered in a common pathway, leading to the activation of the OS mechanism, with the final increase in FRs due to the imbalance in the antioxidant systems’ homeostasis [3]. This imbalance occurs at birth in all newborns as a consequence of the hyperoxic challenge due to the transition from the hypoxic intrauterine environment to extrauterine life [4]. The neonatal brain, especially the preterm neonatal brain, is particularly vulnerable to the insult of OS and sensitive to the dangerous effects of FRs due to the inability to activate the antioxidant defenses [5]. The study and validation of specific and optimal biomarkers of neonatal brain injury, measured in biologic fluids, is an essential step in neuroprotection. These biomarkers could improve the early identification of a newborn at a high risk of brain injury, promoting specific preventive or therapeutic treatment, as the new, modernized standard of care for high-risk neonates [6]. Serum biomarkers could help the clinician to assess the entity of the neonatal brain injury and to monitor the progression of the disease in addition to the standard procedures such as cerebral ultrasound (cUS) and magnetic resonance imaging (MRI). The aim of this educational review is to describe the actual available evidence on serum biomarkers for the early diagnosis of brain damage, analyzing also the causes of neurological diseases for preterm and full-term newborns.

## 2. Brain Development

The second half of gestation and the first 28 days of life are the most important developmental periods for the development of brain structures and cerebral pathways [7]. The neuropathology of brain damage evolves around multiple events that occur at 24–40 weeks of gestation, during the critical stage of cerebral maturation, when cells replication and differentiation are widely activated [8]. Cerebral morphogenesis could be schematically described in 3 steps: (1) histogenesis of the cortex and development of commissures (from 7 to 23 weeks of gestation); (2), cortical morphogenesis and white matter development (from 20 to 40 weeks of gestation); and (3) neuronal myelination (from 18 weeks of gestation to first adult life) [9,10]. Cerebral morphogenesis is finally characterized by an increase in the brain volume, by the development of sulcation, the modification of the ventricular shape, and the reduction in subarachnoid spaces. These processes can be evaluated during the fetal period with the help of fetal brain MRI [8,9]. During this process of maturation, there is also an important neuronal differentiation with synapse formation, the maturation of myelin due to glial cells differentiation, new neurotransmitters synthesis, and vascular development [7,10].

Considering the very complex characteristics of these developing phases, endogenous insults, such as ischemia, inflammation, excitotoxicity, and FRs attack, could affect the normal morphogenesis of brain structures, causing important long-term damages [8]. In addition, it has been largely demonstrated that, in the early phases of life, other events such as under-nutrition, metabolic side effects, drug exposure, and other factors typical of the neonatal intensive care could affects the post-natal cerebral maturation [11,12,13,14,15,16,17]. Considering these dynamic phases of pre-natal and post-natal brain maturation, neonatal life (especially for babies born preterm) is a critical window for long-term neurodevelopment.

## 3. Brain Damage in Preterm Newborn

Preterm birth interrupts the process of cerebral maturation, causing an impaired brain development and increasing the risk for neurodevelopmental delay in postnatal life.

The intraventricular hemorrhage (IVH), produced by an injury of the germinal matrix and the subventricular zone, is one of the leading causes of brain damage in preterm newborns [8]. Intraventricular hemorrhage affects 20–25% of preterm newborns with a birth weight less than 1500 gr [8]. The immaturity of blood vessels in the germinal matrix, a highly vascular cerebral region, combined with poor tissue vascular support, makes this region particularly vulnerable to the development of IVH in preterm newborns [8,18]. In full-term infants, the preferential site of IVH is instead the choroid plexus [7].

**Figure 1:**
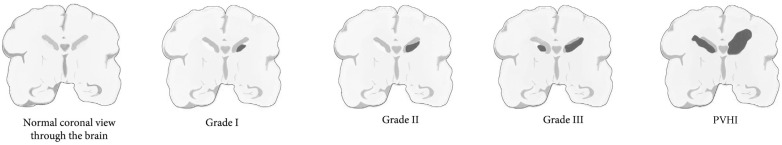
Schematically representation of IVH classification by Volpe. Figure legend: PVHI (periventricular hemorrhagic infarction).

Intraventricular hemorrhage could be classified into three grades according to Volpe (Figure 1) [7]:

- Grade 1: hemorrhage apparently confined to germinal matrix, distended less than 10% of ventricular space;
- Grade 2: hemorrhage clearly localized in the ventricle but occupying 10–50% of ventricular space;
- Grade 3: large hemorrhage, occupying greater than 50% of ventricle with or without ventricular echo-densities.

In addition, when the hemorrhage also includes the parenchymal tissue, the lesion (periventricular hemorrhagic infarction-PVHI) is considered separately because these abnormalities generally are not related simply by the extension of a matrix hemorrhage or IVH into the normal brain parenchyma (Figure 1) [7].

Neonates with IVH could have different clinical presentations that range from an acute deterioration with apnea, pallor, acidosis, hypotension, bulging fontanel, seizures, and decreased muscle tone to a “clinically silent syndrome” when no symptoms are presented [7].

Periventricular leukomalacia (PVL) is another important brain lesion that affects preterm newborns, causing cerebral palsy (CP), cognitive, behavioral, and attention deficits [7]. It is a cerebral white matter injury (WMI) associated with a decreased cortex volume, thalamus, and basal ganglia volume [8]. The lesion starts as a focal lesion that occurs in deep periventricular white matter zones due to neuronal necrosis, reactive gliosis, and microglial activation [7]. Necrosis can be macroscopic (several millimeters or more) and can evolve rapidly in some weeks to multiple cystic lesions, also assessable through a cerebral ultrasound scan (cUS) known as “cystic PVL” [8]. Most commonly, the focal necrosis is microscopic and evolves in several weeks to glial scars which are not visible at the beginning by the neuroimaging [7].

Neuroimaging techniques, such as cUS and MRI, are useful tools for the diagnosis of IVH and PVL [19,20]. However, both present some limitations: cUS is an operator dependent technique, while MRI is not easy to perform during the first weeks of life for the critical clinical condition of the babies and the need for a proper sedation [7,19,20]. Considering the neuroimaging limits and the possible lack of symptoms in the early stage of PVL, it would be useful to identify biomarkers capable of predicting which neonates may suffer from IVH or PVL [7,8]. Despite some studies evaluating the role of innovative neuroprotective therapy (i.e., stem cells, erythropoietin (EPO), vitamin E, and melatonin), the early diagnosis of IVH/PVL is still the gold-standard to reduce a long-term neurological consequence, such as cerebral palsy (CP) [21,22,23].

Cerebral palsy has been defined as “a group of permanent disorders of the development of movement and posture, causing activity limitation, that are attributed to nonprogressive disturbances that occurred in the developing fetal or infant brain” [24]. The prevalence of CP in preterm infants born between 22 and 31 weeks’ gestational age is of 62.5/1000 live births [25]. Despite the fact that a preterm birth is the most important risk factor for CP, the biologic basis seem to be multifactorial [26,27]. There are several perinatal interventions that aim to prevent CP, such as the administration of magnesium sulfate to women at risk of a preterm birth and methylxanthine therapy for apnea of prematurity, essential to reduce the risk of hypoxemia and bradycardia [28,29]. The diagnosis of CP can be made in the first 6 months of age and is based on clinical presentation, in association with other diagnostic tools such as neuroimaging (cUS and MRI) and early standardized motor/neurologic assessments (i.e., Hammersmith Infant Neurological Examinations) [27,30]. Despite the advance in technology in the last decade, the incidence of CP remains higher for the preterm population with a late diagnosis for the efficacy of a neuroprotective intervention. Thus, a rapid screening test, such as serum biomarkers, could be decisive to guarantee an early diagnosis in order to improve the neurological outcome of this higher risk population.

## 4. Brain Damage in Full-Term Newborn

The principal causes of brain damage in full-term newborns are the hypoxic ischemic encephalopathy (HIE), the neonatal stroke, and systemic infections [31]. In the pathogenesis of HIE, the timing of the injury and treatment have fundamental roles [32]. Although the introduction of therapeutic hypothermia as the gold-standard for the treatment of HIE has been an essential change in the prognosis [33,34], this approach can be started only for full-term newborns in the first 6 h of life, and it has only a partial effectiveness because 45% of patients still die or have a neurodevelopmental disability despite this treatment [35]. The first stage of HIE is characterized by a primary energy failure due to hypoxic ischemia (HI), which causes a primary energy failure. This event takes to a decrease in ATP, with an increase in the lactate levels and systemic acidosis [36,37]. Subsequently, the reperfusion of the ischemic zone determines an oxidative metabolism with the activation of inflammation and apoptotic cascades [38]. These events lead to an activation of lipases, proteases, and endonucleases. In particular, lipases induce a production of free fatty acids, especially arachidonic acid, with a release by proteases of superoxide FRs through the activation of cyclooxygenase and the production of prostaglandin [39,40]. This excessive production of FRs during the ischemia/reperfusion process leads to the further production of FRs and other toxic metabolites in larger quantities than the antioxidant capacity of cells. Finally, the third stage after an hypoxic insult, which lasts for months to years, is characterized by reactive gliosis with a persistent inflammation and epigenetic changes [21].

Another cause of near-term brain damage is stroke, involving both arterial and venous cerebral blood vessels, with a hemorrhagic, ischemic, or mixed component [41,42]. The event is symptomatic for 50% of the cases [43]. The most frequent stroke is the arterial ischemic one, but it can be followed by cerebral sinovenous thrombosis and neonatal hemorrhagic stroke [43]. The main clinical manifestation in the first weeks of life are seizures [44,45]. Despite the vulnerability of the zone near to the stroke, endogenous mechanisms, which are still not fully understood, could potentially preserve it [46]. Long term consequences are represented by seizures, CP, congenital hemiplegia, neurodevelopmental delay including intellectual disability, language retardation, and behavioral problems from infancy to adult life [47,48].

Other important causes of brain damage in the neonatal period for full-term newborns, are congenital infections, able to determine a neuronal stem cells injury during the vulnerable phase of brain development. The risk of the transmission of intrauterine during pregnancy is higher with those of an older gestational age, while the risk of adverse fetal consequences significantly increased with the transmission of infection during the first half of pregnancy [49,50]. Considering the TORCH group infections, the congenital cytomegalovirus (cCMV) infection is the primary cause of brain damage in newborns. The incidence of cCMV is higher in low–middle income countries. In addition to neurodevelopmental delay, cCMV is the principal cause of nongenetic sensorial hearing loss [51]. At birth, about 10–15% of congenitally infected infants are symptomatic and approximately half of them will present long-term consequences [52,53]. However, also the 10–15% of asymptomatic infants infected by cCMV could develop long-term sequelae [54]. There is no evidence for the effective treatment of asymptomatic infants and the use of antiviral therapy is contraindicated by an animal model for its toxicity (i.e., neutropenia, thrombocytopenia, gonadal toxicity, and carcinogenicity) [55]. Thus, it is important to find an early biomarker which is able to predict the long-term consequences to justify antiviral therapy in these populations in order to reduce the hearing loss and neurological disability related to CMV infection.

## 5. Serum Biomarkers

**Figure 2:**
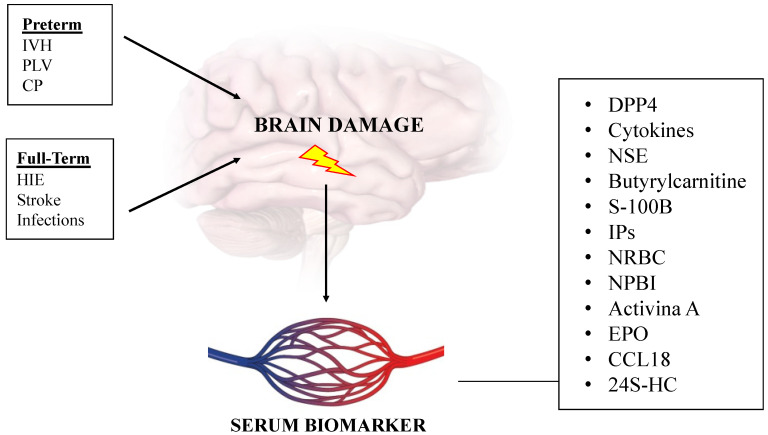
Newborn brain damage and serum biomarkers. Figure legend: IVH (intraventricular hemorrhage); PLV (periventricular leukomalacia); CP (cerebral palsy); HIE (hypoxic ischemic encephalopathy); DPP4 (dipeptidylpeptidase 4); NSE (neuron-specific enolase); S-100B (acidic calcium-binding protein); IPs (F2-isoprostanes); NRBC (nucleated red blood cells); NPBI (non-protein-bound protein); EPO (erythropoietin); CCL18 (chemokine ligand 18); 24S-HC (24S-hydroxycholesterol).

There are many serum biomarkers studied in relation to brain damage for newborns (Figure 2, Table 1):

### 5.1. Dipeptidylpeptidase 4

Dipeptidylpeptidase 4 (DPP4) is a membrane-bound serine protease that is localized in the gastrointestinal tract, liver, lungs, kidneys, and also on T-lymphocytes where the enzyme is known as activation marker CD26. DPP4 and DPP4-like peptidases from the prolyl oligopepetidase family hydrolyze neuropeptides, cytokines, and peptide hormones [80,81,82]. It is supposed that these enzymes could be associated with the neurodegenerative processes related to cerebral ischemia. The participation of DPP4 in the processes of inflammation and neurodegeneration in ischemic brain damage was demonstrated in animals [83].

Yakovleva et al. investigated the serum DPP4 activity in neonates with cerebral ischemia. The serum DPP4 activity in the group of patients with cerebral ischemia was significantly higher than the control group. In addition, preterm and full-term neonates show no differences in DPP4 activity under the influence of hypoxia [56].

Considering the serum DPP4 activity in neonates with different neurological symptomatology: among the full-term neonates with cerebral ischemia, the maximum DPP4 activity was found in the group with an excitement syndrome. These values were significantly higher than those in children with a depression syndrome. Instead, for the preterm infants with cerebral ischemia, there were no statistically significant differences in the serum DPP4 activity between the groups with different functional nervous system activity [56].

It might be supposed that DPP4 takes part in regulating the central nervous system activity in full-term newborns. On the contrary, in preterm newborns, these regulatory mechanisms are still in a developing stage. It is known that DPP4 takes part in the transduction of the signal for the activation of T-helper cells, which leads to their proliferation and cytokine production [84]. Thus, hypoxia could trigger an inflammatory response determining an increase in DPP4 activity in the blood [85,86].

Yakovleva et al. opens new opportunities for the development of DPP4 inhibitors for the prevention of detrimental neurological consequences in newborns with cerebral ischemia [56].

### 5.2. Cytokines

Hypoxic-ischemic injury activates many inflammatory processes in the brain. A number of inflammatory molecules have been suggested to be sentinel biomarkers of HIE. The serum proteins are readily measurable and may be useful biomarkers of injury phases [87].

Microglia are the resident immune cells of the brain, able to start the inflammatory response in the central nervous system [88]. HIE induces microglia cells to produce proinflammatory cytokines, with a damage to the overall structure of the brain [89]. Microglia generate not only an excess of inflammatory cytokines (e.g., TNF-a and IL-1b) but also glutamate, nitric oxide (NO), and reactive oxygen species (ROS), which collectively cause oligodendrocyte death, axonal degeneration, and the disruption of the immature blood–brain barrier [90]. In addition, many inflammatory cytokines have direct toxic effects due to the increased production of inducible NO synthase and cyclooxygenase and the release of FRs [57]. Proinflammatory cytokines might damage developing white matter by inducing intravascular coagulation and/or thrombosis and vasoconstriction or by inducing the production of other cytokines such as the platelet-activating factor [91].

In 2003, Chiesa et al. reported that IL-6 was a good marker of HIE and long-term neurodevelopmental delay [58]. Many years later, Chaparro’s study supported those of Chiesa, showing higher levels of IL-6 in hypoxic patients at birth [57,58]. Moreover, Chaparro et al. demonstrated that the expression of IL-1b and TNF-a was markedly increased by several fold in patients affected by HIE compared with healthy controls, and the IL-6 expression was significantly increased by nine-fold [57].

A Greek study, in agreement with more recent studies, showed that the asphyxiated neonates had significantly higher IL-6 and IL-1ß serum levels than healthy controls. On the contrary, the TNF-a serum levels did not differ between the two groups of neonates studied (asphyxiated neonates and healthy controls) [59]. No differences in the serum levels of TNF-a were found between the neonates who showed neurologic abnormalities and those without neurodevelopmental delay [59].

### 5.3. Neuron-Specific Enolase

Neuron-specific enolase (NSE) is a highly specific glycolysis isoenzyme for neurons and peripheral neuroendocrine cells [92].

The NSE level was measured as a marker of neuronal death. Clinical studies showed that the NSE concentrations are significantly increased in HIE and asphyxiated neonates compared with the healthy controls [57,61,62,63]. A significant rise in NSE in term infants with HIE seems to be associated with conspicuous neurological damage or death. Chaparro et al. revealed that the NSE level in the blood of neonates may be correlated with the severity of encephalopathy and brain injury [57]. The other three trials confirmed these results, while Nagdyman et al. found no differences in the level of NSE in 29 asphyxiated neonates compared with 20 controls [60,61,62,63].

### 5.4. Butyrylcarnitine

Fatty acid b-oxidation takes place in the mitochondria, involving at least 31 enzymes or the transporters involved. Short-chain acyl-CoA dehydrogenase (SCAD) is the enzyme that catalyzes the first phase of mitochondrial fatty acid β-oxidation. Increased butyrylcarnitine is the result of the dysfunction of this enzyme [93].

A retrospective observational cohort study by Lopez-Suarez et al. analyzed the acetyl-carnitine profile of 67 infants with early HIE in the perinatal period (days 1–7 of life) [62].

The acetyl-carnitine profile is generally measured in neonatal screening for the early detection of inherited metabolic disease by means of electrospray tandem ionization with mass spectrometry coupled to a high-performance liquid chromatography system [94,95].

Lopez-Suarez et al. found a significant positive correlation between butyrylcarnitine and NSE [62]. Therefore, butyrylcarnitine and NSE seems to be the best prognostic biomarkers of neuronal insult in HIE.

### 5.5. Acidic Calcium-Binding Protein

The acidic calcium-binding protein (S-100B) is a member of a family of calcium binding proteins named S-100 proteins [96]. This protein is localized to some extent in non-neuronal cell types including melanocytes, Langerhans cells, dendritic cells in lymphoid organs, chondrocytes, Leydig cells, adrenal medulla satellite cells, and skeletal muscle satellite cells [97]. S-100 proteins work as calcium sensor proteins that modulate biological activity via calcium binding and perform several cellular mechanisms within the cell populations that contain it such as necrosis and apoptosis [98,99,100]. Additionally, S100B is found in extracellular biological fluids, due to its active secretion from cells [98].

Focusing on the central nervous system, S-100B is expressed and released by astrocytes. This secretion by glia is an early response to metabolic injury (i.e., oxygen, serum, and glucose deprivation) and could be released in biological fluids at an early stage with a renal metabolism [87,101]. The S100B half-life is about one hour. Cord blood and urine seems to be a perfect source to titrate the S100B concentration using LIAISON or ELISA techniques within 2–6 h [68,102].

A number of studies evaluated the ability of S-100B testing to predict brain injury. In this context, Beharier’s study summarizes the available data regarding the sensitivity and specificity values of S100B testing in urine and serum (monitoring time points up to 24 h), showing a sensitivity of 50% to 73% and a specificity of 74% to 90% in serum [97].

This protein plays a trophic role during the development of the nervous system with paracrine/autocrine/endocrine trophic role at low, physiological concentrations (nanomolar). These trophic effects include neuronal survival, muscle development, and the regeneration and promotion of neurite extension [97,103]. However, its overexpression can have dangerous effects due to the activation of inducible NO synthase and the subsequent production of NO with astrocyte cell death [104]. Since the S-100B protein, during an active brain injury, is released from a damaged tissue into circulation, its concentration increases at an early stage of hypoxia in both cerebrospinal fluid and cord blood. For this reason, the best sources for biomarkers are the fluids obtained the least invasively and shortly after birth [105]. Chaparro et al. collected white blood cells isolated from venous blood from children with encephalopathy, using polymerase chain reaction analysis that revealed substantial increases in the expression of S-100B by 97% compared with healthy controls [57]. Serum concentrations of S-100B increase in correlation with the severity of HIE, white matter brain lesions, and this has long-term neurological consequences: in severe asphyxia, the S-100B levels were found to be high immediately after birth and continue to rise with time (up to one week); in mild asphyxia, the blood levels exhibit only a slight elevation soon after birth and decline from this point further with time [61,67,106,107,108,109,110]. Newborns with no signs of asphyxia, instead, present baseline levels of the protein at all times [96].

The data revealed that S-100B testing can discriminate immediately after birth between asphyxiated newborns with a severe clinical outcome and newborns suffering from asphyxia with no clinical outcome. The ability of S100B testing to predict prognosis was in association with the severity of the cases [109]. However, S-100B predictive values have been shown to be less accurate in cases where the clinical outcome is severe but not clearly defined [70]. Interestingly, a marked elevation of the protein was found also in term and preterm asphyxiated newborns complicated with IVH [64,66,107].

Previous studies about S100B concentrations that increased in the case of a brain injury suggested that higher S100B levels in pregnancies with FGR reflect fetal chronic hypoxia [65,106,111]. So, the protein can be used as a biomarker of brain damage in growth-restricted newborns [65,69]. The examination of the cord blood S-100B concentration may be helpful in identifying SGA newborns at a higher risk of postnatal neurological sequelae at an early stage in cases where a prenatal Doppler examination is normal, even when the standard clinical and laboratory parameters are silent, and an early stage neurologic follow-up is uneventful [68]. Gazzolo et al. reported that higher concentrations of S-100B were detected in the mother of FGR fetuses who developed IVH after birth [112]. To satisfy the criteria for the reliable use of S-100B as an accurate screening test, combinations of biomarkers might be needed to improve the outcome prediction. For example, combining S-100B with NSE, a biomarker of early neuronal necrotic damage, may increase the early detection of neuronal damage and expose different patterns of brain damage [60,61,63,96].

### 5.6. F2-Isoprostanes

F2-Isoprostanes (IPs) are made by the peroxidation of lipids in cell membranes as the result of FRs-induced injury [113]. So, IPs are a useful biomarker for lipid peroxidation. They can be important in clinical practice because preterm white matter is vulnerable to a lipid peroxidation-mediated injury [72].

In preterm infants, during the developmental window between 23 and 32 weeks, the brain is at a significant risk of WMI: the white matter can be exposed to OS due to hypoxia-ischemia [114]. Preoligodendrocytes (OLs) dominate the white matter during this phase [115] and appear particularly prone to FRs-mediated injury because of the immaturity of antioxidant defenses, whereas differentiated OLs are more resistant to OS [116].

Quantifying the IPs in human brain tissue, it can be possible to identify in the glia the aldehydes which originated from lipid hydroperoxides [116].

Coviello et al. showed that the cord blood Ips (cb-Ips) and plasma Ips (pl-Ips) (between 24 and 48 h after birth) levels were not significantly different [72]. Univariate regression analysis demonstrated that cb-IPs were not associated with WMI at the term of an equivalent age and with the cognitive and motor outcome at 24 months of the corrected age. Instead, pl-IPs were positively associated with WMI at the term of an equivalent age; especially, a higher pl-IPs concentration and lower GA were associated with a higher WMI score. In this study, the pl-IPs levels plotted curve indicated that 31.8 pg/mL had the best predictive threshold, with a sensitivity of 86% and a lower specificity of 60% to discriminate newborns with and without WMI, while they were not associated with the cognitive and motor outcome at 24 months of a corrected age [72]. Matthews et al. demonstrated increased pl-IPs in preterm infants at risk of severe abnormalities on neuroimaging [71]. These results revealed that the early pl-IPs concentration was higher in infants with WMI and this correlation remained statistically significant after adjusting for potential confounding factors. Thus, the pl-IPs levels might be a valuable early biomarker of WMI [72]. In addition, Coviello et al. demonstrated a relation between higher pl-IPs levels and a decreased functional brain activity measured with amplitude-integrated EEG [73]. These data are in accordance with the literature according to which IPs are significantly raised in infants with WMI [116].

### 5.7. Nucleated Red Blood Cells

Another marker of brain damage which has been described is the rise in nucleated red blood cells (NRBC) in the peripheral neonatal blood following birth asphyxia [74]. Fotopoulos’s study showed that at a mean postnatal age of 24 h, the absolute numbers of NRBC in the peripheral blood of asphyxiated neonates were significantly higher compared with those of the controls, while during the following days, the absolute NRBC numbers showed a progressive fall in both groups [59]. In addition, in the same period, the NRBCs numbers were significantly higher in the neonates who developed neurologic abnormalities than in those who had a normal neurologic development [59]. Florio et al. confirmed these results [75].

### 5.8. Non-Protein-Bound Protein

The term non-protein-bound protein (NPBI) indicates a form of iron free from plasma protein binding and with a low molecular mass. The method for measuring the NPBI levels in small samples is based on the preferential chelation of NPBI by a large excess of low affinity ligand nitrilotriacetic acid through high performance liquid chromatography [117]. Free iron is toxic when not bound to proteins because it is potentially available to produce the hydroxyl radical by reacting with H_2_O_2_ through the Fenton reaction, the latter being the main cause of oxidative damage [118]. Brain damage is caused by the absorption of plasma NPBI, which crosses the damaged blood–brain barrier. The high concentrations of plasma NBPI serve the oligodendrocytes in the process of differentiation, thus increasing their susceptibility to OS from FRs. Additionally, the hypoxia and ischemia caused by perinatal asphyxia strongly contributes to the release of NPBI [4,119].

Buonocore et al. showed that no children with normal NBPI values subsequently exhibited neurological abnormalities, showing a 100% sensitivity and 100% specificity for a good neurological outcome. Additionally, a high concentration of NPBI in addition to an exposure to high amounts of lipids leads to the formation of IsoPs, which also cause oxidative damage [76].

### 5.9. Activina A

Activin is a member of the transforming growth factor β superfamily, a trophic factor that regulates the differentiation and proliferation of a wide variety of cells [120]. Activin A is a neuroprotective factor during brain damage and hypoxic-ischemic damage. Mechanical irritation and chemical brain damage evoke a strong upregulation of activin A [121,122]. Activin A and its receptors are widely distributed throughout the brain. High plasma concentrations of Activin A have been found in both IVH premature infants and term infants with moderate to severe asphyxia, in which cases the activin A analysis reached a sensitivity of 100% and a specificity of 93% as a single marker [75,77,123].

### 5.10. Erythropoietin

EPO is a glycoprotein hormone produced mainly by the kidneys in response to cellular hypoxia [124,125]. EPO and its receptor are also expressed in astrocytes, neurons, and endothelial cells of the brain [126].

The pilot study by Bhandari et al. demonstrated that high concentrations of EPO were present in the umbilical cord blood of 116 infants under 34 weeks of age with IVH, diagnosed by cUS [78]. The advantage of measuring the EPO concentration in the blood is that it can be measured at birth and the results are available the same day.

### 5.11. Chemokine Ligand 18

Chemokine ligand 18 (CCL18) is encoded on chromosome 17q11.2 and belongs to the CC chemokine family. It plays a key role in the lymphocyte homing and primary immune response and the CCL18 receptor is detectable in the choroid plexus, periventricular capillary endothelium, ependymal cells, and germline matrix [127].

Therefore, inflammatory conditions can lead to the increased plasma levels of CCL18 [128]. Premature infants who developed CP and patients with traumatic injuries have been shown to have lower levels of CCL18 in umbilical cord blood in the first case and elevated levels of CCL18 in brain tissue biopsies in the second [79,129].

Kallankari et al. measured in 116 premature infants less than 107 umbilical cord blood immunoproteins. Infants who developed IVH shortly after birth had lower concentrations of umbilical cord chemokine CCL18 than very preterm babies without IVH [79]. CCL18 seems to predict the risk of grade II-IV IVH, having ruled out its association with chorioamnionitis or funisitis. High levels of CCL18 block the action of the agonist ligands on CCR3 and thus inhibit the degranulation of leukocyte and, consequently, the inflammatory activity acts as a protective factors against IVH and brain injury [79].

### 5.12. 24S-Hydroxycholesterol

24S-hydroxycholesterol (24S-HC) is a brain-derived cholesterol metabolite, produced by neuron-specific cytochrome P450 enzyme, CYP46A1, exclusively in the brain and it is capable of crossing the blood–brain barrier into circulation and is excreted in bile [130]. CYP46 converts cholesterol into 24S-HC via hydroxylation and upregulates the cholesterol efflux through the activation of the nuclear transcription factor X. It has been demonstrated that CYP46 is responsible for cholesterol efflux in the brain [131].

Recent works, in animal models, suggested that 24S-HC might be a promising novel lipid biomarker for the extent of HI brain injury [132,133]. A few trials have demonstrated the effects of neonatal encephalopathy on brain cholesterol synthesis and the regulation of the cholesterol metabolism, suggesting not only that its metabolites (specifically those released from the brain into the serum) may potentially act as biomarkers to aid in the identification or severity stratification of hypoxic–ischemic brain injury, but also that the cholesterol pathways may be therapeutic in neonatal encephalopathy [132,133].

The hypothesis of using 24S-HC as an HI brain injury marker is made for several reasons: the most important one is that the HI-induced upregulation of CYP46A1 (in mouse) mediates the increased formation of brain 24S-HC, leading to its elevation in the blood at 6 h and 24 h after HI, suggesting that one mechanism resulting in the decreased cholesterol levels may be cholesterol efflux [132,134].

The activation of CYP46A1 leading to an increase in 24SHC could be due to the effects of increased glutamate and OS, as a result of excitotoxicity, because it has been reported that they can enhance the promoter activity of CYP46A1 [135,136,137].

Fuxin et al. demonstrated that serum 24S-HC could be an acute marker of an HI brain injury if measured within 24 h after the insult (cell death responses peak), but it is of limited value if it is measured beyond this time window [138]. In clinical practice, when the pregnant patients show evidence of acute peripartum or intrapartum hypoxia or the interruption of the placental blood flow, the serum 24S-HC can be measured within 24 h to evaluate the brain injury and provide early prognostic information [138]. The 24S-HC levels can be used in conjunction with other criteria for selecting babies eligible for therapeutic hypothermia within 6 h from their birth [138]. According to these studies in animal models, 24S-HC can be considered an early predictive serum marker for both a diagnostic and prognostic application for infants with HIE.

### 5.13. Neurotrophins

The nerve growth factor (NGF) and brain-derived neurotrophic factor (BDNF) are crucial for the development of the peripheral and central nervous system [139]. A growing body of evidence suggests that neurotrophic factors can protect neurons against neuronal death [140,141]. Kirschner et al. examined whether the systemic administration of members of the neurotrophin family, NGF, BDNF, neurotrophin 3, and neurotrophin 5, and the basic fibroblast growth factor (bFGF) could protect against brain damage in neonatal rats [142]. It was demonstrated that neurotrophins and bFGF can attenuate hypoxia-induced neuronal damage by reducing OS.

In an experimental model of hypoxic-ischemic brain damage, inflammatory cytokines such as TNF-α, ICAM-1, and IL-1β contribute to neuronal apoptosis, whereas neurotrophins NGF and BDNF antagonize it [143].

Recently, urinary NGF has been suggested as an early prognostic indicator of a high long-term risk of motor and cognitive impairment in SGA and preterm neonates [144].

## 6. Placenta and Perinatal Brain Damage

The placenta plays a key role in the plasticity of fetal development. Fetal neurodevelopment is the mirror of the chemical, biochemical, and placental histopathological composition; therefore, placental examination can be a useful tool to identify fetal/neonatal brain damage early and assess its extent [2]. The inflammation and alteration of placental perfusion are associated with higher levels of OS biomarkers in umbilical cord blood. This may indicate an increased fetal susceptibility to oxidative damage [145].

Normal placental functions can be altered by the abundant production of ROS and reactive nitrogen species. Cord blood and the placenta represent important sources of OS biomarkers that can consent to identify early high-risk newborn [2]. Perrone et al. tested the hypothesis that a placental injury is associated with increased levels of OS biomarkers in umbilical cord blood. They analyzed the three different OS markers of isoprostanes, non-protein-bound iron, and advanced oxidative protein products and they found that these markers were detectable in the umbilical cord blood in patients born to mothers with chorioamnionitis and reduced perfusion [145]. The disadvantage of fetal biomarkers is that they do not allow for follow-up studies because more samples are needed in order to monitor the changes throughout time.

Yanni et al. demonstrated that placental inflammation and postnatal systemic inflammation together were associated with a higher risk for white matter damage according to the two-hit hypothesis [146]. The first hit is placental inflammation and the second hit is the elevated concentration of the cytokines (CRP, TNF-α, IL-8, and ICAM-1) in the top quartile for gestational and postnatal age. The synergetic action of the two factors was associated with ventriculomegaly, hypoechoic lesion, and microcephaly [146].

The inflammation of the placenta was also associated with low corticotropin-releasing hormone (CRH) mRNA concentrations. The high activity of placental CRH (identical in structure, immunoreactivity, and bioactivity to hypothalamic CRH) determines much of the increase in free cortisol of the pregnant woman [147,148]. Cortisol is extremely important for brain development in an anatomical and functional sense and very low levels are associated with poor neurodevelopment; although, very high levels negatively affect brain development [149].

Leviton et al. explained that the placenta CRH concentration appears to convey information about the risk of brain damage in extremely preterm newborns. They showed that infants whose placenta had a low concentration of CRH mRNA were at an increased risk of cUS abnormalities such as ventriculomegaly [150].

Stressful events during pregnancy may adversely affect brain development and may increase the risk of neurodevelopmental disorders later in life. It has been assumed that early changes in the kynurenine pathway of tryptophan degradation, which contains quinolinic acid, a neuroactive metabolite, may represent the molecular link between prenatal stress and delayed pathological consequences [151].

All together, these data suggest that endogenous prenatal phenomena influence the risk of brain damage in preterm infants. Further studies on the postnatal evaluation of human placental cellular and molecular mediators could be useful for identifying unfavorable intrauterine conditions, the early stratification of a population of newborns at risk for brain damage, and developing successful intervention/prevention strategies.

## 7. Conclusions

Not only preterm but also the term brain is particularly vulnerable to the OS-related damage. The presence of an association between biomarkers of oxidative stress measured in the first hours of life and brain damage (successfully evaluated through neuroimaging) emphasizes the possibility of an early identification of newborns at a greater risk of brain damage.

Reliable early specific serum markers, associated with brain damage in newborns, are currently lacking in clinical practice. S-100B seems to be the most studied biomarker. However, the literature is inconsistent due to the complexity of brain damage etiology, the timing of the insult, and blood sampling, limiting the validation for its use in clinical practice. An ideal biomarker, to be useful, should inform about the condition at the time of the measurement for an early treatment and predicts disease progression in order to provide patients with more information on the future clinical outcomes. The ideal biomarker, furthermore, should be easily isolated from the blood, with a low cost, and identify in infants with a brain injury early in the first few hours of their life. In addition, a good biomarker may help to identify the timing of the injury; this is essential because an HI injury often begins in utero, and if too much time has elapsed since the initial brain injury, the neonate will not benefit from treatment. Cut off values are needed to move the biomarkers from the bench to the bedside and the assay must be not only of a high sensitivity but suitable for a very rapid processing and return of the results for the clinician to act on. Finally, the correlation between placental inflammation and brain damage should be evaluated in further studies. It might be useful to identify a panel of placental biomarkers capable of identifying patients at risk of brain damage earlier than neonatal serum biomarkers. For the best prognosis, more studies should focus also on the association of these biomarkers with the type and severity of perinatal brain damage considering the low number of studies that evaluate this relation.

## Figures and Tables

**Table 1:** Summary of the studies that evaluated the relation between serum biomarkers and brain damage in newborns.

Biomarker	Reference	Population	Sample Source (i.e., Cord or Peripheral Blood)	Brain Damage Patterns
DPP4	Yakovleva et al. [56]	39 full-term and 40 preterm	Cord and peripheral blood (3–5 days of life and before discharge)	↑ cerebral ischemia
Cytokines	Chaparro-Huerta et al. [57]	62 full-term newborns (32 control vs. 30 Asphyxiated)	Plasma samples in the first 24 h of life	TNF-α, IL-1β and IL-6 ↑ in HIE
	Chiesa et al. [58]	Full-term: 50 case and 113 controls	Cord blood	IL-6 ↑ in HIE and worse neurodevelopmental outcome
	Fotopoulos et al. [59]	57 LBW newborns	Peripheral blood	TNF-α, IL-1β and IL-6 ↑ Asphyxiated/infected
NSE	Chaparro-Huerta et al. [57]	62 full-term newborns (32 control vs. 30 Asphyxiated)	Plasma samples in the first 24 h of life	↑ in HIE
	Nagdyman et al. [60]	29 asphyxiate and 20 control infants	cord blood and plasma samples at 2, 6, 12, and 24 h after birth	No difference
	Giuseppe et al. [61]	30 neonates with perinatal asphyxia and 10 control	Capillary blood	↑ in Perinatal Asphyxia
	López-Suárez et al. [62]	67 full-term newborns with HIE, 827 full-term control	Serum samples collected in the first 6 h of life and on the 2nd and 3rd day of life	↑ in HIE
	Celtik et al. [63]	91 full-term newborns	Peripheral blood	↑ in HIE
Butyrylcarnitine	López-Suárez et al. [62]	67 full-term newborns with HIE, 827 full-term control	Serum samples collected in the first 6 h of life and on the 2nd and 3rd day of life	↑ in HIE
S-100B	Nagdyman et al. [60]	29 asphyxiated and 20 control infants	Cord blood and plasma samples at 2, 6, 12, and 24 h after birth	↑ in HIE
	Giuseppe et al. [61]	30 neonates with perinatal asphyxia and 10 control	Capillary blood	↑ in Perinatal Asphyxia
	Gazzolo et al. [64]	29 full-term newborns with IVH, 20 asphyxiated infants without IVH, and 80 normal newborns.	Plasma sample at 12 h of life	↑ in IVH
	Gazzolo et al. [65]	10 IUGR with normal and 10 with abnormal umbilical artery doppler findings. 40 uncomplicated pregnancies	Cord blood	↑ in IUGR, positive correlation with middle cerebral artery pulsatility index and with umbilical artery pulsatility index to middle cerebral artery pulsatility index ratio.
	Gazzolo et al. [66]	11 IVH and 14 controls	Plasma samples in the first 72 h of life	↑ in IVH, correlated with the grade of hemorrhage. Positive correlation with the middle cerebral artery pulsatility index and S-100B.
	Murabayashi et al. [67]	22 normal and 40 newborns with brain diseases	Serum samples on day 1, 2 and 6	↑ in HIE and Asphyxia
	Chaparro-Huerta et al. [57]	62 full-term newborns (32 control vs. 30 Asphyxiated)	Plasma samples in the first 24 h of life	↑ in HIE
	Strzalko et al. [68]	88 SGA vs. 80 AGA	Cord blood	↑ in SGA
	Velipaşaoğlu et al. [69]	32 IUGR vs. 29 controls	Cord blood	↑ in IUGR
	Thorngren-Jerneck et al. [70]	62 full-term infants with birth asphyxia	Serum samples in the first 2–3 days of life	↑ in relation with the grade of HIE and ↑ in CP
IPs	Matthews et al. [71]	136 preterm ≤ 28 wks	Plasma samples on days 14 and 28 of life	↑ in worse developmental outcomes at 12 months
	Coviello et al. [72]	44 preterm < 28 wks	Cord and peripheral blood (24–48 h of life)	↑ in WMI
	Coviello et al. [73]	39 preterm < 28 wks	Cord and peripheral blood (24–48 h of life)	↑ in poor EEG activity
NRBC	Green et al. [74]	149 preterm	Peripheral blood first 6 days of life	↑ IVH
	Fotopoulos et al. [59]	57 LBW newborns	Peripheral blood	↑ Asphyxiated/infected
	Florio et al. [75]	50 preterm	Cord blood	↑ Hypoxic
NPBI	Buonocore et al. [76]	384 newborns (225 >36 weeks, 90 from 32 to 36 weeks and 69 <32 wks).	Cord blood	↑ in worse neurodevelopmental outcome
Activina A	Florio et al. [75]	50 preterm	Cord blood	↑ Hypoxic
	Florio et al. [77]	53 preterm	Arterial blood in the 1st h of life	↑ IVH
EPO	Bhandari et al. [78]	116 preterm	Cord blood	↑ IVH
CCL18	Kallankari et al. [79]	163 preterm <32 wks.	Cord blood	↓ IVH
24S-HC		No study in human babies, only animals.		
NGF	Aisa et al.	43 preterm >32 wks and full-term neonates.	No plasma or serum, only urine.	↓ in worse developmental outcomes at 24 months

Table legend: DPP4 (dipeptidylpeptidase 4); HIE (hypoxic ischemic encephalopathy); NSE (neuron-specific enolase); S-100B (acidic calcium-binding protein); IUGR (intrauterine growth restriction); IVH (intraventricular hemorrhage); SGA (small for gestational age); AGA (adequate for gestational age); CP (cerebral palsy); IPs (F2-isoprostanes); WMI (white matter injury); NRBC (nucleated red blood cells); NPBI (non-protein-bound protein); EPO (erythropoietin); CCL18 (chemokine ligand 18); 24S-HC (24S-hydroxycholesterol), NGF (nerve growth factor). ↑: increase; ↓: decrease.

## Abbreviations

OS	oxidative stress
FRs	free radicals
cUS	cerebral ultrasound
MRI	magnetic resonance imaging
IVH	intraventricular hemorrhage
PVHI	periventricular hemorrhagic infarction
PVL	periventricular leukomalacia
CP	cerebral palsy
HIE	hypoxic ischemic encephalopathy
HI	hypoxic ischemia
cCMV	congenital cytomegalovirus
DPP4	dipeptidylpeptidase 4
NO	nitric oxide
ROS	reactive oxygen species
NSE	neuron-specific enolase
SCAD	short-chain acyl-CoA dehydrogenase
S-100B	acidic calcium-binding protein
SGA	small for gestational age
AGA	adequate for gestational age
IPs	F2-Isoprostanes
WMI	white matter injury
OLs	preoligodendrocytes
NRBC	nucleated red blood cells
NPBI	non-protein-bound protein
EPO	erythropoietin
CCL18	chemokine ligand 18
24S-HC	24S-hydroxycholesterol
NGF	nerve growth factor
BDNF	Brain-derived neurotrophic factor
bFGF	basic fibroblast growth factor
CRH	corticotropin-releasing hormone
